# Diagnostic Values of miR-21, miR-124, and M-CSF in Patients With Early Cervical Cancer

**DOI:** 10.1177/1533033820914983

**Published:** 2020-05-01

**Authors:** Fang Ruan, Yun-fei Wang, Yun Chai

**Affiliations:** 1 Department of Gynecology, Affiliated Hospital of Jining Medical College, Jining, Shandong, China

**Keywords:** miR-21, miR-124, M-CSF, cervical cancer, diagnostic value

## Abstract

**Objective:**

This study aimed to investigate the diagnostic values of microRNA-21, microRNA-124, and macrophage colony-stimulating factor in patients with cervical cancer.

**Methods:**

A total of 68 patients with cervical cancer admitted in our hospital (cervical cancer group) and 57 healthy individuals undergoing physical examinations (healthy group, also control group) were enrolled in this study. The expression of serum microRNA-21 and microRNA-124 was detected by quantitative reverse transcription polymerase chain reaction. The expression of serum macrophage colony-stimulating factor was detected by enzyme-linked immunosorbent assay. The diagnostic values of microRNA-21, microRNA-124, and macrophage colony-stimulating factor in cervical cancer were analyzed. The correlations between the expression of microRNA-21 and microRNA-124 with that of macrophage colony-stimulating factor were also analyzed.

**Results:**

Compared to those in the healthy group, patients in the cervical cancer group had a higher expression of microRNA-21 and macrophage colony-stimulating factor (*P* < .05) but lower expression of microRNA-124 (*P* < .05). The expression of microRNA-21, microRNA-124, and macrophage colony-stimulating factor in the patients correlated with the tumor size, tumor node metastasis (TNM) staging, tumor differentiation, and the presence or absence of lymph node metastasis and human papillomavirus infection (*P* < .05). According to the receiver operating characteristic curves, the area under the curve of microRNA-21 for diagnosing cervical cancer was 0.723, the specificity was 58.82%, and the sensitivity was 91.23%. The area under the curve of microRNA-124 was 0.766, the specificity was 94.12%, and the sensitivity was 57.89%. The area under the curve of macrophage colony-stimulating factor was 0.754, the specificity was 64.71%, and the sensitivity was 87.72%. Pearson correlation analysis showed that the expression of microRNA-21 positively correlated with that of macrophage colony-stimulating factor (*r* = 0.6825, *P* < .001), and the expression of microRNA-124 negatively correlated with that of macrophage colony-stimulating factor (*r* = −0.6476, *P* < .001).

**Conclusion:**

MicroRNA-21, microRNA-124, and macrophage colony-stimulating factor may be involved in the development and progression of cervical cancer. The detection of serum microRNA-21, microRNA-124, and macrophage colony-stimulating factor has good sensitivity and specificity in the diagnosis of cervical cancer.

## Introduction

Cervical cancer is the second most common cancer after breast cancer.^1^ Its mortality in the low- and middle-income countries is reported to be 18 times higher than that in the high-income countries.^2^ One of the major risk factors for cervical cancer is persistent high-risk human papillomavirus (HPV) infection. Clinically, most patients with cervical cancer are in the middle and advanced stages when treated, and thus, they miss the best time window for treatment.^3^ Therefore, molecular biomarkers related to the early diagnosis of cervical cancer should be evaluated, and the regulatory mechanisms of molecular signals during the development and progression of the disease should be explored. Moreover, therapeutic targets and biomarkers that affect the development and progression should be discovered. All of these are important for improving the early diagnosis and treatment of the disease as well as for improving the prognosis of the disease.^4,5^

MicroRNAs (miRNAs) are widely present in eukaryotic cells; they are highly conserved and endogenous noncoding, 19- to 25-nucleotide-long transcripts.^6,7^ Some miRNAs are closely related to the development and progression of cervical cancer. For instance, Tan *et al* have found that miR-378 enhances the migration of cervical cancer by directly targeting the autophagy-related protein 12.^8^ After overexpressing miR-375 in the human cervical cancer cells, SiHa and CaSki, Wang *et al* discovered that miR-375 inhibits the migration of the cells through the target gene *SP1*.^9^ Hu *et al* have shown that miRNA-200a and miRNA-9 inhibit the metastasis of cervical cancer cells and predict the survival time of patients with cervical cancer.^10^ These studies indicate that miRNAs may play an important role in the development and progression of cervical cancer and can be used as biomarkers for the early diagnosis of this disease. MicroRNA-21 (miR-21) is highly expressed in gastric cancer, prostate cancer, and breast cancer; therefore, it is considered a carcinogenic miRNA.^11-13^ Previous studies have confirmed that microRNA-124 (miR-124) has a low expression in malignant tumors, such as gastric cancer, and is associated with the formation and malignant progression of some tumors, such as breast cancer.^14,15^ According to Sun *et al*, low expression of miR-124 correlates with the poor prognosis of pancreatic cancer.^16^ As a special cytokine existing in the bone marrow cavity, macrophage colony-stimulating factor (M-CSF) promotes macrophage colony formation and regulates the proliferation and differentiation of macrophages.^17^ A study has shown that M-CSF plays an important role in the tumors and is involved in the proliferation of tumor cells and tumor angiogenesis.^18^ However, the roles of miR-21, miR-124, and M-CSF in cervical cancer have been rarely studied.

At present, the gold standard for the diagnosis of cervical cancer is a cervical biopsy, which has difficulties in obtaining specimens and arriving at a timely diagnosis.^19^ The acquisition of the blood tumor markers is relative simple, of which the operation is easy to automate, the detection results are quantitative data, and the test price is low; hence, it is easy to be applied in the early screening. Therefore, the expression of serum miR-21, miR-124, and M-CSF in patients with cervical cancer was explored in this study. The diagnostic values of these markers in cervical cancer, as well as the correlation among these 3 biomarkers, were analyzed to provide a reference for the application of these potential molecular biomarkers in cervical cancer.

## Materials and Methods

### General Information

A total of 68 patients with cervical cancer admitted to our hospital (cervical cancer group) and 57 healthy individuals undergoing physical examinations (healthy group, also control group) from May 2014 to January 2015 were enrolled in this study. The patients in the cervical cancer group were aged between 45 and 59 years, with a mean age of 47.57 ± 8.19 years. The individuals in the healthy group were aged between 43 and 61 years, with a mean age of 48.39 ± 10.17 years. The inclusion criteria were patients confirmedly diagnosed with cervical cancer by liquid-based ThinPrep cytology test (TCT), HPV testing, and B-mode ultrasound of the reproductive system. The healthy individuals underwent physical examinations in the physical examination center of our hospital and had normal examination results. All of them had no other tumors or cardiac, hepatic, and renal diseases. Their family members had no history of cancer. The exclusion criteria were pregnant women, patients with thyroid diseases and immunological diseases, patients who were bedridden for a long time, patients with severe hypertension and diabetes, patients who had taken glucocorticoids and antibiotics in the preceding 2 weeks, and patients with mental and cognitive disorders. All patients did not receive radiotherapy, chemotherapy, or other treatment before blood collection. The study was approved by the ethics committee of Affiliated Hospital of Jining Medical College. All study participants provided written informed consent before participating in the study.

### Main Instruments and Reagents

Macrophage colony-stimulating factor enzyme-linked immunosorbent assay (ELISA) kit (YM-QP10199; Shanghai YuanMu Biological Technology Co., Ltd., Shanghai, China), a multifunctional ELISA microplate (Infinite 200 PRO; Coslan scientific LTD, Guangzhou, China), Light Cycler real-time fluorescence quantitative PCR instrument (Roche, Basel, Switzerland), total RNA extraction kit (Solarbio R1200; Shanghai Hengfei Refrigeration Engineering Equipment Co., Ltd., Shanghai, China), M-MLV reverse transcription kit (Vazyme, Nanjing, China), ultraviolet spectrophotometer (Multiskan Sky; Thermo Fisher, Shanghai, China), qReal-time PCR kit (Invitrogen, Grand Island, New York), and SYBR Green qPCR Master Mix kit (Thermo Fisher) were obtained. The primers for miR-21, miR-124, and the internal reference (U6) were synthesized by Sangon Biotech Co, Ltd. (Shanghai, China, Table 1).

**Table 1.** Primers for miR-21, miR-124, and U6.

Genes	Forward Primers	Reverse Primers
miR-21	5′-GCTTCGCCTAGCTTA TCAGACT-3′	5′-CAGTGCTGGGTCCG AGTGA-3′
miR-124	5′-GCTAAGGCACGCGG TG-3′	5′-GTGCAGGGTCCGAG GT-3′
U6	5′-CTCGCTTCGGCAGC ACA-3′	5′-AACGCTTCACGAAT TTGCGT-3′

Abbreviations: miR-21, microRNA-21; miR-124, microRNA-124.

### Detection Method

Fasting venous blood samples (5 mL) were obtained from the study participants belonging to the 2 groups in the morning. The samples were placed in vacuum blood collection tubes and centrifuged at 3000*g* per minute for separation. Macrophage colony-stimulating factor was detected by ELISA, while miR-21 and miR-124 were detected by quantitative reverse transcription polymerase chain reaction (qRT-PCR) strictly according to the kit instructions. The optical density values at 450 nm were measured within 30 minutes.

#### Quantitative Reverse Transcription Polymerase Chain Reaction

The total RNA was extracted from the serum according to the instructions mentioned in the TRIzol extraction kit. The synthesized cDNA was stored at −20°C for later use. The system (20 µL in total) was as follows: 10 µL of PCR Premix, 2 µL of upstream primer (10×; concentration of 10 μmol/L), 2 µL of downstream primer (10×; concentration of 10 μmol/L), and double-distilled water (RNase- and DNase-free, finally added to make up to 20 μL). The ABI PRISM 7500 (Shanghai PuDi Biotech Co.,Ltd., Shanghai, China) fluorescence quantitative PCR instrument manufacturer software was used to analyze the amplification data. The results were expressed by 2^−△△CT^.^20^

### Statistical Methods

SPSS 21.0 was used for the statistical analyses. The categorical variables data were expressed by n (%). The continuous variables were expressed by mean ± standard deviation. The continuous variables between the groups were compared by *t* test, whereas the categorical variables were compared by χ^2^ test. Receiver operating characteristic (ROC) curves were plotted to assess the diagnostic values of miR-21, miR-124, and M-CSF in cervical cancer. Pearson correlation coefficient was used to analyze the correlations between miR-21, miR-124, and M-CSF. *P* < .05 was considered statistically significant.

## Results

### General Information

There were no statistically significant differences between the cervical cancer and healthy groups in terms of age, body mass index, history of smoking, history of drinking, past medical history, exercise habits, place of residence, blood glucose, alanine aminotransferase, aspartate aminotransferase, hemoglobin, red blood cell count, and platelet count (*P* > .05; Table 2).

**Table 2.** General Information.^a^

Categories	Cervical Cancer Group (n = 68)	Healthy Group (n = 57)	t/χ^2^	*P* Value
Age (years)	47.57 ± 8.19	48.39 ± 10.17	0.499	.618
BMI (kg/m^2^)	19.27 ± 3.06	19.68 ± 2.76	0.780	.437
History of smoking			0.182	.669
Yes	19 (27.94)	14 (24.56)		
No	49 (72.06)	43 (75.44)		
History of drinking			0.118	.732
Yes	21 (30.88)	16 (28.07)		
No	47 (69.12)	41 (71.93)		
Past medical history			0.289	.866
Hypertension	8 (11.76)	7 (12.28)		
Diabetes	3 (4.41)	4 (7.02)		
Hyperlipidemia	4 (5.88)	5 (8.77)		
Exercise habits			0.435	.509
Yes	39 (57.35)	36 (63.16)		
No	29 (42.65)	21 (36.84)		
Place of residence			0.468	.494
City	58 (85.29)	46 (80.70)		
Countryside	10 (14.71)	11 (19.30)		
Glu (mmol/L)	5.97 ± 0.46	6.03 ± 0.39	0.778	.438
ALT (U/L)	21.06 ± 9.13	22.42 ± 9.51	0.814	.417
AST (U/L)	19.28 ± 7.06	18.43 ± 7.56	0.649	.518
Hb (g/dL)	14.47 ± 0.93	14.51 ± 0.87	0.247	.806
RBC (×10^12^/L)	4.28 ± 0.46	4.24 ± 0.38	0.524	.602
PLT (×10^9^/L)	153.76 ± 18.35	155.08 ± 19.37	0.391	.697

Abbreviations: ALT, alanine aminotransferase; AST, aspartate aminotransferase; BMI, body mass index; Glu, glucose; Hb, hemoglobin; PLT, platelet; RBC, red blood cell.

^a^ The values are represented as n (%) or mean ± standard deviation.

### Correlations Between miR-21, miR-124, and M-CSF With the Clinicopathological Features

The expression of miR-21, miR-124, and M-CSF in patients with cervical cancer correlated with the tumor size, TNM staging, tumor differentiation, and the presence or absence of lymph node metastasis and HPV infection (*P* < .05). However, it did not correlate with age, place of residence, and tumor types (*P* > .05; Table 3).

**Table 3.** Correlations of miR-21, miR-124, and M-CSF With the Clinicopathological Features.^a^

Factors	n	miR-21	*t*/*F*	*P* Value	miR-124	*t*/*F*	*P* Value	M-CSF (pg/mL)	*t*/*F*	*P* Value
Age (years)			0.150	.881		0.910	.366		0.318	.751
≤50	38	4.12 ± 1.28			1.02 ± 0.34			419.14 ± 141.19		
>50	30	4.07 ± 1.46			1.09 ± 0.28			408.34 ± 135.84		
Place of residence			0.054	.957		0.382	.704		0.374	.710
Countryside	21	4.10 ± 1.34			1.08 ± 0.34			418.64 ± 144.51		
City	47	4.08 ± 1.44			1.05 ± 0.28			405.26 ± 132.59		
Tumor types			0.004	.996		1.125	.331		0.013	.987
Squamous cell carcinoma	32	4.11 ± 1.25			1.12 ± 0.29			408.48 ± 135.86		
Adenocarcinoma	25	4.08 ± 1.37			1.04 ± 0.34			414.15 ± 137.48		
Others	11	4.10 ± 1.48			0.97 ± 0.27			412.33 ± 128.65		
Tumor size (cm)			6.833	<.001		4.658	<.001		5.829	<.001
≤4	41	3.11 ± 1.28			1.31 ± 0.38			335.64 ± 128.21		
>4	27	5.41 ± 1.47			0.92 ± 0.26			532.94 ± 148.49		
TNM staging			8.769	<.001		5.935	<.001		6.857	<.001
Stages I + II	43	2.94 ± 1.15			1.36 ± 0.36			322.45 ± 112.62		
Stages III + IV	25	5.80 ± 1.52			0.88 ± 0.24			538.63 ± 144.95		
Tumor differentiation			3.515	.001		3.706	<.001		4.418	<.001
Moderate highly differentiated	44	3.67 ± 1.22			1.28 ± 0.36			355.95 ± 134.67		
Poorly differentiated	24	4.97 ± 1.82			0.96 ± 0.30			511.26 ± 145.49		
Lymph node metastasis			4.919	<.001		5.855	<.001		5.537	<.001
No	40	3.41 ± 1.48			1.37 ± 0.40			346.36 ± 122.55		
Yes	28	5.16 ± 1.39			0.87 ± 0.25			525.28 ±142.62		
HPV infection			4.119	<.001		4.577	<.001		4.385	<.001
No	25	3.65 ± 1.31			1.31 ± 0.37			357.26 ± 145.19		
Yes	43	5.17 ± 1.55			0.94 ± 0.29			510.97 ± 135.94		

Abbreviations: *F*, the statistical value of *F* test; HPV, human papillomavirus; M-CSF, macrophage colony-stimulating factor; miR-21, microRNA-21; miR-124, microRNA-124; *t*, the statistical value of *t* test; TNM, tumor node metastasis.

^a^ Values are represented as mean ± standard deviation.

### Comparison of Expression of miR-21, miR-124, and M-CSF

**Figure 1.**
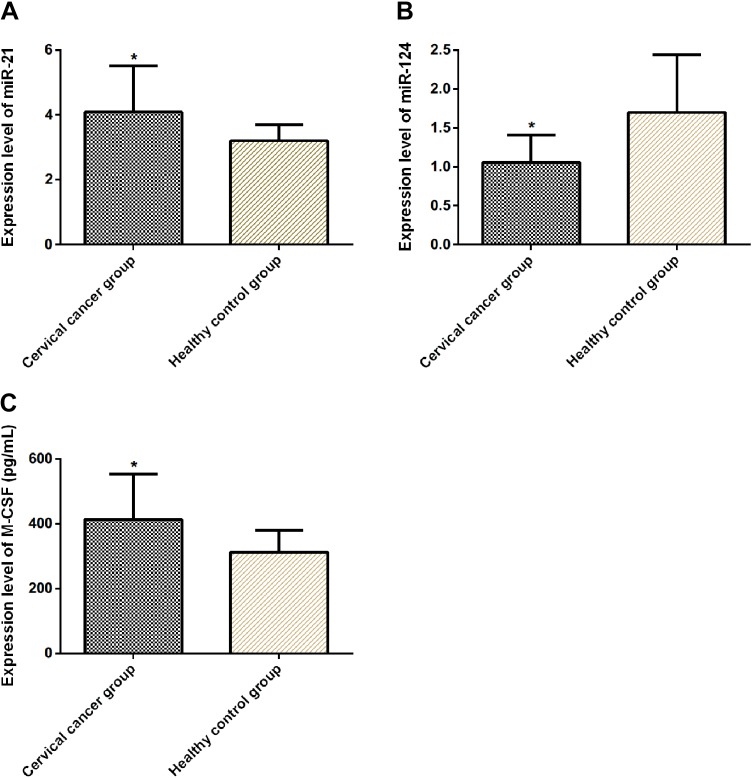
Comparison of the expression of miR-21, miR-124, and M-CSF. The expression of miR-21 in the cervical cancer group was higher than that in the healthy group (*P* < .05) (A). The expression of miR-124 in the cervical cancer group was lower than that in the healthy group (*P* < .05) (B). The expression of M-CSF in the cervical cancer group was higher than that in the healthy group (*P* < .05) (C). *P < .05 as compared to the healthy group. M-CSF indicates macrophage colony-stimulating factor; miR-21, microRNA-21; miR-124, microRNA-124.

Compared to individuals in the healthy group, patients in the cervical cancer group had higher expression of miR-21 and M-CSF (*P* < .05) but lower expression of miR-124 (*P* < .05; Table 4, Figure 1).

**Table 4.** Comparisons of the Expression of miR-21, miR-124, and M-CSF.^a^

Groups	n	miR-21	miR-124	M-CSF (pg/mL)
Cervical cancer group	68	4.09 ± 1.43	1.06 ± 0.35	413.65 ± 140.77
Healthy group	57	3.20 ± 0.50	1.70 ± 0.74	312.23 ± 68.51
*t*	-	4.473	6.339	4.966
*P*	-	<.001	<.001	<.001

Abbreviations: M-CSF, macrophage colony-stimulating factor; miR-21, microRNA-21; miR-124, microRNA-124; *t*, the statistical value of *t* test.

^a^ Values are represented as mean ± SD.

### Diagnostic Values of miR-21, miR-124, and M-CSF

**Figure 2.**
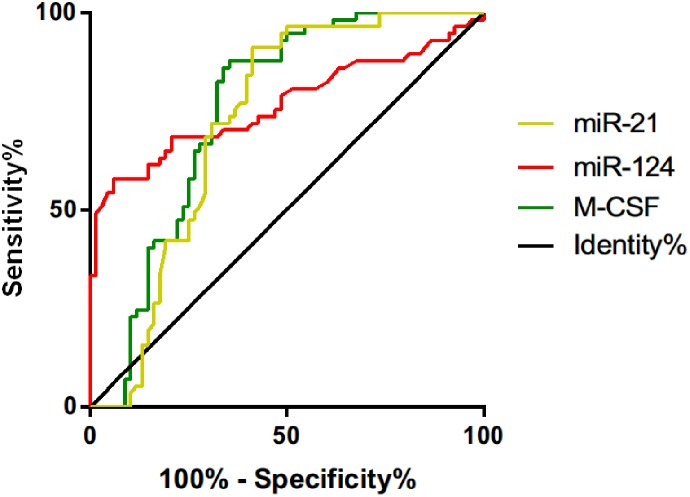
Diagnostic values of miR-21, miR-124, and M-CSF. The AUC of miR-21 for diagnosing cervical cancer was 0.723 (95% CI: 0.631-0.815), the specificity was 58.82%, the sensitivity was 91.23%, and the cutoff value was 3.855. The AUC of miR-124 was 0.766 (95% CI: 0.677-0.856), the specificity was 94.12%, the sensitivity was 57.89%, and the cutoff value was 1.67. The AUC of M-CSF was 0.754 (95% CI: 0.666-0.841), the specificity was 64.71%, the sensitivity was 87.72%, and the cutoff value was 382.70 pg/mL. AUC indicates area under the curve; CI, confidence interval; M-CSF, macrophage colony-stimulating factor; miR-21, microRNA-21; miR-124, microRNA-124.

According to the ROC curves, the area under the curve (AUC) of miR-21 for diagnosing cervical cancer was 0.723 (95% confidence interval [CI]: 0.631-0.815), the specificity was 58.82%, the sensitivity was 91.23%, and the cutoff value was 3.855. The AUC of miR-124 was 0.766 (95% CI: 0.677-0.856), the specificity was 94.12%, the sensitivity was 57.89%, and the cutoff value was 1.67. The AUC of M-CSF was 0.754 (95% CI: 0.666-0.841), the specificity was 64.71%, the sensitivity was 87.72%, and the cutoff value was 382.70 pg/mL (Table 5, Figure 2).

**Table 5.** Diagnostic Values of miR-21, miR-124, and M-CSF.

Indicators	AUC	95% CI	Specificity (%)	Sensitivity (%)	Cutoff Value
miR-21	0.723	0.631-0.815	58.82 (40/68)	91.23 (52/57)	3.855
miR-124	0.766	0.677-0.856	94.12 (64/68)	57.89 (33/57)	1.67
M-CSF	0.754	0.666-0.841	64.71 (44/68)	87.72 (50/57)	382.70 pg/mL

Abbreviations: AUC, area under the curve; CI, confidence interval; M-CSF, macrophage colony-stimulating factor; miR-21, microRNA-21; miR-124, microRNA-124.

### Correlations Between miR-21 and miR-124, and M-CSF

**Figure 3.**
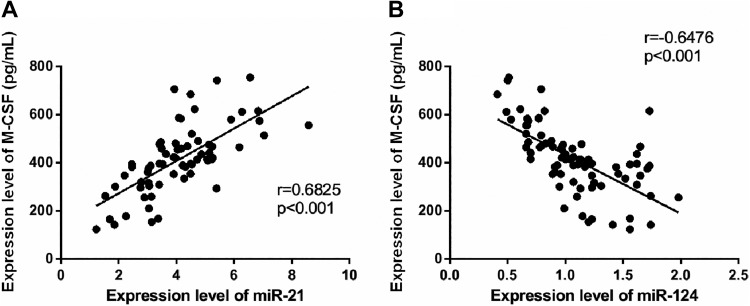
Correlations between miR-21 and miR-124 and M-CSF. The expression of miR-21 positively correlated with that of M-CSF (*r* = 0.6825, *P* < .001) (A). The expression of miR-124 negatively correlated with that of M-CSF (*r* = −0.6476, *P* < .001) (B). M-CSF indicates macrophage colony-stimulating factor; miR-21, microRNA-21; miR-124, microRNA-124.

According to the Pearson correlation analysis, the expression of miR-21 positively correlated with that of M-CSF (*r* = 0.6825, *P* < .001) and the expression of M-CSF increased with that of miR-21. The expression of miR-124 was negatively correlated with that of M-CSF (*r* = −0.6476, *P* < .001), and the expression of M-CSF decreased with that of miR-124 (Figure 3).

## Discussion

Cervical cancer is a disease related to viral infection.^21^ Although the incidence of cervical cancer in the developed countries have reduced due to the screening of the disease and use of HPV vaccines, the incidence of the disease in the undeveloped countries has not decreased and the overall survival rate has not increased.^22,23^ Therefore, it is of great significance for the prevention and treatment of cervical cancer to understand the mechanisms of development and progression of the disease.^24^

With progress in research, it has been found that HPV infection alone cannot cause the malignant transformation of the cervical cells and that changes in the functions and expression of other genes are also involved in the pathological process of cervical cancer.^25,26^ According to a recent study, as a new research hotspot in tumor biology, miRNA has been closely related to the development, progression, invasion, metastasis, and apoptosis of tumors, functioning as an oncogene or a tumor suppressor gene.^27^ Previous studies have shown that miR-21 and miR-124 are abnormally expressed in cervical cancer. After analyzing HPV16-positive CaSKi cells using clonal sequencing, Wang *et al* found that miR-21 is one of the miRNAs that has the most significant difference in expression.^28^ According to Wang *et al*, miR-124-3p inhibits the metastasis of cervical cancer cells by directly targeting IGF2BP, and its reduced expression is associated with advanced cervical cancer.^29^ However, previous studies on the expression of miR-21 and miR-124 in cervical cancer were limited to the exploration of cervical cancer cell lines cultured *in vitro*, and no studies were conducted on the expression of miR-21 and miR-124 in clinical patients with cervical cancer. Macrophage colony-stimulating factor is the major cytokine that regulates the differentiation and growth of monocyte–macrophage cell lines.^30^ Recent studies have found that its expression abnormally increases in many tumors and that circulating M-CSF in the serum has become a molecular marker for these tumors.^31,32^ These findings indicate that M-CSF plays an important role in the development and progression of many tumors. In this study, compared to those in the healthy group, patients in the cervical cancer group had higher expression of miR-21 and M-CSF but lower expression of miR-124. The expression of the 3 markers correlated with the tumor size, TNM staging, tumor differentiation, and the presence or absence of lymph node metastasis and HPV infection. These findings suggest that miR-21, miR-124, and M-CSF may be involved in the development and progression of cervical cancer. According to Yao *et al*, miR-21 is highly expressed in rapidly proliferating cervical cancer Hela cells, and inhibition of its expression inhibits cell proliferation.^33^ Wilting *et al* found methylation of miR-124 in the detection of cervical cancer cell lines,^34^ indicating that gene silencing caused by the hypermethylation of the coding gene miR-124 may be involved in the development of cervical cancer. According to Baghdadi *et al*, the expression of M-CSF is related to poor survival of patients with lung cancer. Macrophage colony-stimulating factor activates the colony-stimulating factor 1 receptor, which plays a pivotal role in many aspects related to the tumor microenvironment.^35^ According to Van *et al*, differences in the M-CSF receptor signal transduction regulate monocyte maturation and macrophage polarization in the tumor microenvironment.^36^ We speculate that the abnormal expression of M-CSF in cervical cancer may be correlated with the regulation of the tumor microenvironment, but the specific mechanism has not been explored in this study. We further discussed the diagnostic value of miR-21, miR-124, and M-CSF in cervical cancer. The results showed that the AUC of miR-21 for diagnosing the disease was 0.723, the specificity was 58.82%, and the sensitivity was 91.23%. The AUC of miR-124 was 0.766, the specificity was 94.12%, and the sensitivity was 57.89%. The AUC of M-CSF was 0.754, the specificity was 64.71%, and the sensitivity was 87.72%. These findings suggest that these 3 biomarkers have certain value in the diagnosis of patients with cervical cancer. Clinically, these 3 biomarkers can be used to screen patients with high-risk cancer having cervical disease. In addition, the sensitivity of miR-21 and M-CSF is relatively high, but the specificity level is relatively low, while miR-124 shows the opposite. Cervical cancer screening methods include Pap smear, TCT, second-generation gene hybridization capture technology, colposcopy, cervical tissue biopsy, and so on. However, the collection of serum samples is more convenient and quick, which is convenient for initial screening and will not cause great trauma to patients. Clinically, feasibility and ease of use can be considered according to the situation, and these 3 can be combined with other existing diagnostic methods, for example, screening for these markers in women with abnormal cytology results or other patients at high risk of malignant cervical disease, or for whom routine cervical cancer screening procedures are not available, and so on, which can help diagnose and judge diseases more quickly and accurately in the early stage, so as to provide the basis for clinical treatment. Finally, according to the Pearson correlation analysis, the expression of miR-21 positively correlated with that of M-CSF, while the expression of miR-124 negatively correlated with that of M-CSF. This indicates that the expression of miR-21 and miR-124 in serum may be closely related to M-CSF, which, however, was not fully explored in this study.

This study retrospectively analyzed the expression of miR-21, miR-124, and M-CSF in the serum of patients with cervical cancer and healthy individuals and discussed the diagnostic value of their combined detection in cervical cancer. However, there are some limitations. It is difficult to avoid the selection bias by using the patients already diagnosed with cervical cancer as the test subjects. Moreover, the different expression levels of stage II and stage III/IV may indicate different diagnostic performance for the detection of stage I/II and more advanced diseases. However, the sample size may be lower due to such subgroup analysis. These deficiencies need to be supplemented in future studies.

In summary, miR-21, miR-124, and M-CSF may be involved in the development and progression of cervical cancer. The detection of serum miR-21, miR-124, and M-CSF has good sensitivity and specificity in the diagnosis of cervical cancer.

## Abbreviations

AUC: area under the curve
M-CSF: macrophage colony-stimulating factor
miRNA: microRNA
miR-21: microRNA-21
miR-124: microRNA-124
HPV: human papillomavirus
qRT-PCR: quantitative reverse transcription-polymerase chain reaction
ROC: receiver operating characteristics
TCT: ThinPrep cytology test.
